# Dietary Available Phosphorus Affected Growth Performance, Body Composition, and Hepatic Antioxidant Property of Juvenile Yellow Catfish *Pelteobagrus fulvidraco*

**DOI:** 10.1100/2012/987570

**Published:** 2012-08-02

**Authors:** Qin Tang, Chunfang Wang, Congxin Xie, Jiali Jin, Yanqing Huang

**Affiliations:** ^1^College of Fisheries, Huazhong Agricultural University, Wuhan 430070, China; ^2^East China Sea Fisheries Research Institute, Chinese Academy of Fishery Sciences, Shanghai 200090, China

## Abstract

An 8-week feeding trial was carried out with juvenile yellow catfish to study the effects of dietary available phosphorus (P) on growth performance, body composition, and hepatic antioxidant property. Six pellet diets were formulated to contain graded available P levels at 0.33, 0.56, 0.81, 1.15, 1.31, and 1.57% of dry matter, respectively. Triplicate tanks with each tank containing 60 juveniles (3.09 ± 0.03 g) were fed one of the six experimental diets for 8 weeks. Specific growth rate, feeding rate, and protein efficiency ratio were significantly higher at 0.81% dietary available P. Efficiency of P utilization distinctly decreased with increasing P level. Body lipid content significantly decreased while body ash and feces P content significantly increased with increasing P level. Quadratic regression analysis indicated that vertebrae P content was maximized at 1.21% dietary available P. Fish fed 1.57% dietary available P had highest activity of hepatic superoxide dismutase and catalase and malonaldehyde content. In conclusion, decreasing dietary available P increased P utilization efficiency and body lipid content while decreased vertebrae P content. Juvenile yellow catfish were subjected to oxidative damage under the condition of high dietary P content (1.57%), and the damage could not be eradicated by their own antioxidant defense system.

## 1. Introduction

Phosphorus (P) is an important constituent of nucleic acids and cell membranes and is directly involved in all energy-producing cellular reactions [1, 2]. Fish must obtain P from their food because of the low concentration of phosphate in natural waters [3]. P deficiency or P excess could probably affect the production of ATP, the synthesis of nucleic acids, and the complement of cell membranes, thus cause anorexia, transient lethargy, reduced growth, and dark coloration [4]. In most fish, the main signs of P deficiency include poor growth, poor feed efficiency, and bone mineralization [1]. 

Phosphorus deficiency enhanced lipid peroxidation and induced oxidative stress in plants [5], such as, bean [6], barley [7], and oilseed rape [8]. With the decrease of inorganic phosphate concentration in tissues, lipid peroxidation increased in cells [6]. Normally, generation of reactive oxygen species (ROS) is kept under control by antioxidant defense system that includes antioxidative enzymes, superoxide dismutase (SOD), catalase (CAT), peroxidase (POD), and glutathione peroxidase (GPX) and enzymes of Halliwell-Asada pathway and antioxidative molecules: ascorbates, *α*-tocopherol, carotenoids, flavonoids, and glutathione [9–11]. Overproduction of ROS results in oxidative stress, a deleterious process that can be an important mediator of damage to cell structures, including lipids and membranes, proteins, and DNA [12]. In the process of eliminating the free radicals, SOD, GSH-Px, and CAT are the major indirect free radical scavengers [12]. In unfavorable circumstance, the balance between the activities and the intracellular levels of these antioxidants is broken, and the imbalance will result in the accumulation of malonaldehyde (MDA) and finally lead to oxidative damage to cell [13]. 

Therefore, appropriate P is critical for organisms to maintain both structural and functional roles and to form an intricate and interwoven system regulating the healthy functioning [5]. P starvation is an abiotic stress that imposes an oxidative stress in plant root cells [6]. However, there was no clear report about the relationship between dietary P deficiency or P excess and oxidative responses in animals. Three types of metabolic control of oxidative metabolism observed in the various living tissues of animals had been reported: in skeletal muscle by ADP (or Pi/phosphocreatine), in cardiac tissue a coordinated control of substrate delivery that may involve Ca^2+^ and inorganic P control of NADH, and control of O_2_ delivery [14]. Also, in rheumatoid arthritis patients, oxidative metabolism within the cell had been altered and oxygen free radicals were generated, which altered calcium and phosphorus levels in skeletal muscle cells [15]. 

Yellow catfish (*Pelteobagrus fulvidraco*) is a freshwater species native to Asian-Pacific waters, which is now becoming one of the major commercial species cultured by farmers in China. The annual production of yellow catfish reached 163,556 tons in the year of 2009, an increase of 21.65% over the year 2008 [16]. Most farmers use commercial feed to produce high output. However, these feeds are usually high in phosphorus content (about 20 to 25 kg Ca(H_2_PO_4_)_2_·H_2_O per ton feed). Large amounts of uneaten feed and feces released into the aquatic environment lead to eutrophication and cause a serious threat to the sustainable aquaculture of this species. P is the limiting nutrient for algae growth in freshwater ecosystem [17]. The effect of P is immediate and broad, causing both environmental concern and possible economic inefficiencies in the production systems due to increased costs associated with the biological and chemical processing of the waste [1].

Therefore, a need exists to determine the effects of dietary P content on fish performance and health functioning in the aquaculture setting, to balance the desire for high fish production with protection of the aquatic environment. Little research on dietary P, especially hepatic antioxidant property under dietary P stress, has been conducted on yellow catfish. In the present study, six diets were formulated to contain low to high levels of P by adding Ca(H_2_PO_4_)_2_·H_2_O into the basal diet. Ca(H_2_PO_4_)_2_·H_2_O was chosen as the source of P due to two reasons: first, reports showed that dietary calcium concentration or calcium-phosphorus (Ca/P) imbalance has no effect on P requirement of catfish [18–20]; second, in the same growth trial, the expression of NaPi-IIb cotransporter was needed to be detected in the intestine, which transports inorganic P from lumen to blood when dietary P content is pretty low in lumen (in another manuscript) and probably would be affected if we chose NaH_2_PO_4_·H_2_O or KH_2_PO_4_·H_2_O. 

The purpose of this study was to investigate the effects of different dietary P levels on growth performance, body composition, and hepatic antioxidant property of juvenile yellow catfish.

## 2. Materials and Methods

### 2.1. Diet Preparation

Six experimental diets were formulated to contain graded levels of available P (0.33, 0.56, 0.81, 1.15, 1.31, and 1.57 g 100 g^−1^ feed, resp., and alpha cellulose used as filler). Formulation and chemical composition of the diets are presented in Table 1. P content in each ingredient was as follows (as % dry matter): casein 0.74, blood meal 0.44, gelatin 0.08, squid meal 0.97, and wheat flour 0.01. Chromic (III) oxide (Cr_2_O_3_) was added (1.0%) to the diets as an inert digestion marker.

All the ingredients were finely ground to pass through 60 mesh sieve (250 *μ*m) and weighed. The dry ingredients were mixed one by one manually for at least 20 min. Then fish oil and soy bean oil were added and mixed-in for another 20 min. Finally the mixture was transferred to a mixer and homogenized for about 20 min. Distilled water was added to the mixture to achieve a proper pelleting consistency, and then the mixture was extruded through a 2.0 mm diameter die to form pellets. After that, diets were dried in an oven at 60°C until the moisture was reduced to less than 10%, then stored in a freezer at −20°C until use. A representative sample was taken for proximate analysis. The stability of P in diets was tested by measuring the total P content in the diets before and after immersed in water for 30 min, and the result showed that the experimental diets held 97.35% total P after immersed in water for 30 min.

### 2.2. Growth Trial

About 1400 yellow catfish larvae were obtained from Southlake hatchery based in Wuhan (Hubei, China) and then were stocked in 18 indoor fiberglass tanks (water volume, 395 L) in aquaculture center of The College of Fisheries, Huazhong Agricultural University. Before starting the experiment, all the larvae were acclimatized to laboratory conditions for four weeks; for the first two weeks, the larvae were fed with Tubificidae and the next two weeks were fed with the basal diet (diet 1) (Table 1). Prior to the feeding trial, all fish were starved for 24 h. Altogether 1080 fish with similar size (3.09 ± 0.03 g) were distributed into 18 indoor fiberglass tanks (395 L) at a stocking density of 60 fish per tank. Fifty fish with similar size around 3 g were anaesthetized in MS 222 (Sigma) solution (100 mg L^−1^) and then kept at −20°C as initial samples for proximate analysis. Each diet was fed for eight weeks (from August to October in 2010) to triplicate groups of fish. During the experiment, water flow was maintained constant at 1.5 L min^−1^, water temperature was 27 ± 0.4°C, dissolved oxygen was about 8 mg L^−1^, and pH was 7.84 ± 0.03. During the experiment, the diurnal cycle was 12-h light/12-h dark.

Every morning about one hour before feeding, about 25% of the water from each tank was drained and replaced with fresh water. Feed was distributed to fish at 08:00 and 16:00 to apparent satiation, and each feeding period lasted for about 2 h. The fish were fed every 30 min and altogether 4 times for each feeding period. Since yellow catfish was active in feed intake, this feeding strategy was to ensure fish in each tank eating up almost all the feed in about 30 min. Apparent satiation was assessed by the feeding behavior of yellow catfish: when we threw the feed into the tanks, fish were not interested in the feed and did not swim quickly to get the feed actively. Uneaten feed was collected after the fourth feeding during each feeding period and complete feces were collected by siphoning after 2 h of feeding from the second week. The uneaten feed was dried to constant weight at 60°C in an oven and weighed to determine daily feed consumption. Pooled feces of each tank were also dried at 60°C. Mortality was checked daily. The cultured water was disinfected periodically by chlorine dioxide to prevent diseases. 

### 2.3. Sample Collection and Analysis

At the end of the experiment, all fish were starved for 24 h, counted, bulk weighed by tank for the calculation of survival rate and growth rate, and then returned to each tank. Ten fish were randomly selected from each tankanaesthetized in MS 222 (Sigma) (200 mg L^−1^) and then kept at −20°C for whole body proximate composition and mineral analysis. 

Five fish from each tank were selected randomly, anaesthetized (MS 222, 200 mg L^−1^), and then kept at −20°C for vertebrae samples. Vertebrae were removed from fish after heating fish in a microwave oven for 120 s, then lightly scrubbed and washed with distilled water to remove surrounding tissues and muscles, dried in an oven at 105°C for 6 h, extracted with 20 mL chloroform and methanol (1 : 1, v/v) for 12 h to remove lipid, and then dried. 

Three fish from each tank were selected randomly, anaesthetized (MS 222, 200 mg L^−1^) first, and then liver samples of these three fish from each tank were frozen immediately in liquid nitrogen and kept at −80°C until use. Before measuring the activity of antioxidative enzymes, the pooled livers by tank were manually homogenized in a glass homogenizer with 0.7% NaCl (w/v) to 10% homogenate. 

Chemical compositions of the dried whole fish, experimental diets, pretreated vertebrae and feces were examined using the following procedure: body ash content and vertebrae ash content by incinerating samples at 550°C for 18 h in a muffle furnace, then wet digested with HCl and HNO_3_ subsequently for the analysis of P content by the molybdovanadate method [21]; crude protein (N × 6.25) by the Kjeldahl method [21]; crude lipid by extraction with petroleum ether for 12 h in a Soxhlet extractor [21]; moisture content by drying to constant weight at 105°C for 6 h [21]. Body calcium and vertebrae calcium contents were analyzed by atomic absorption spectrophotometer (Varian AA240FS Fast Sequential AA Spectrometer, USA). Chromium content of experimental diets and feces was determined using the procedure of Bolin et al. [22]. 

Activities of liver superoxide dismutase (SOD), catalase (CAT), and glutathione peroxidase (GSH-Px) and content of malonaldehyde (MDA) were all analyzed spectrophotometrically using diagnostic reagent kits (Nanjing Jiancheng Bioengineering Institute, China). 

### 2.4. Calculation and Statistical Analysis

Growth parameters were calculated according to the following equations:

(1) Specific  growth  rate  (SGR  %  day−1) =100×[Ln(final  weight)−Ln(initial  weight)]duration  (days),Feed  efficiency  (FE  %)=100×wet  weight  gaindry  feed  intake,Feeding  rate  (FR  %  BW  day−1) =100×dry  feed  intake[days×(FBW+IBW)/2],Survival  (%)=100×final  numbers  of  fishinitial  numbers  of  fish,Protein  efficiency  ratio  (PER  %)=weight  gaincrude  protein  intake,Protein  retention  efficiency  (PRE  %) =100×(protein  retained  in  fish  bodyprotein  intake),Apparent  digestibility  coefficient  of  dry  matter  (ADCd  %) =100×[1−(Cr2O3  in  the  dietCr2O3  in  the  feces)],Apparent  digestibility  coefficient  of  protein  (ADCp %) =100×[1−(Cr2O3 in  the  dietCr2O3  in  the  feces)     ×(crude  protein  in  fecescrude  protein  in  the  diet)],Apparent  digestibility  coefficient  of  P  (ADCph  %) =100×[1−(Cr2O3  in  the  dietCr2O3  in  the  feces)×(P  in  fecesP  in  the  diet)],Efficiency  of  phosphorus  (P)  utilization  (%) =100×[(final  phosphorus  fish  content)phosphorus  intake     − (initial  phosphorus  fish  content)phosphorus  intake],Available P content of the diets=total P content×apparent digestibility coefficient of  P (ADCph) of the diets.

Since we wanted to compare the levels of the dietary available P to see if change in the levels leads to a significant change in the response, we chose the model of one-way analysis of variance (ANOVA) to analyze all the relative responses. When differences among levels were identified, Duncan's multiple-range tests were used to determine the significant differences among different level treatment means of dietary available P at *α* = 0.05 (Statistica 8.0 software, StatSoft Inc, Tulsa, Oklahoma). The data were presented as means ± SE. 

To estimate maximum vertebrae P, we fitted a quadratic regression line to vertebrae P as a function of dietary available P level using SPSS 16.0 (SPSS Inc, Chicago, Ill, USA).

## 3. Results

### 3.1. Apparent Digestibility, Efficiency of P Utilization, and Feces P

Apparent digestibility coefficient of P (ADCph), dry matter and protein digestibility was shown in Table 2. There was no significant difference in dry matter and protein digestibility (ADCd and ADCp) (*P* > 0.05) among all treatments. Apparent digestibility of P (ADCph) significantly increased with increasing dietary available P up to the 0.81% level (*P* < 0.05) and then plateaued with further increases in dietary available P. Efficiency of P utilization decreased (*P* < 0.05) and feces P content increased with the increase of the dietary P level (Table 2).

### 3.2. Growth Performance

Effects of dietary available P on growth performance were shown in Table 3. The final body weight (FBW), specific growth rate (SGR), and feeding rate (FR) were all significantly improved by dietary available P up to 0.81%  (*P* < 0.05) and then significantly decreased compared to the 0.81% dietary group (*P* < 0.05). The protein efficiency ratio (PER) of the 0.81% and 1.57% groups was significantly higher than that of the other groups (*P* < 0.05). However, dietary available P had no significant effects on feed efficiency (FE), protein retention efficiency (PRE), and survival rate.

### 3.3. Body Composition

**Figure 1:**
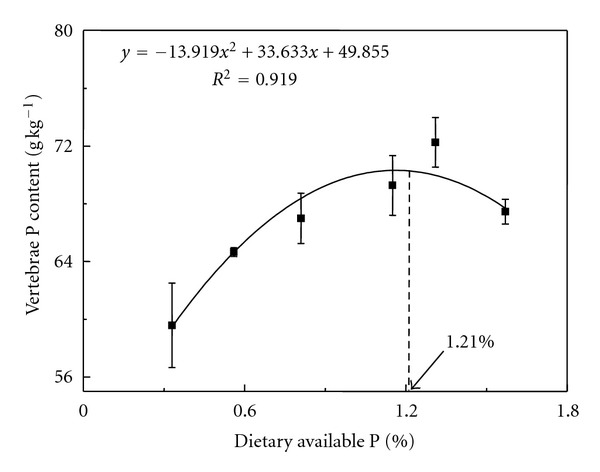
Effect of dietary available P level on vertebrae P content of juvenile yellow catfish. The predicted maximum level occurred at dietary available P equal to 1.21%, based on quadratic regression analysis.

Dietary available P level significantly affected yellow catfish whole body crude lipid, ash, Ca, vertebrae P, Ca, and ash content (*P* < 0.05) (Table 4). With the increase of dietary available P, body crude lipid content significantly decreased (*P* < 0.05). Body ash, vertebrae ash, and vertebrae P content increased until available P reached 1.31%  (*P* < 0.05), then decreased. Quadratic regression analysis showed that the maximum vertebrae P accumulation occurred at a dietary available P of 1.21% (Figure 1).

### 3.4. Antioxidant Property

Dietary available P content significantly affected hepatic SOD and CAT activity and MDA content (*P* < 0.05) (Table 5). Fish in the highest dietary available P content (1.57%) group had higher SOD, CAT activity, and MDA content than all the other groups (*P* < 0.05). There was no significant difference in GSH-Px activity among all the groups (*P* > 0.05).

## 4. Discussion

In the present study, we found the efficiency of P utilization increased with decreasing dietary available P. There are two patterns for P absorption in animals. When the concentration of inorganic P is high in the lumen, passive transportation of P is predominant; when the concentration of P is low, active transportation of P via NaPi cotransporter is predominant [23]. The present study showed that lower dietary P level led to higher P utilization; similar results were observed in rainbow trout [24] and black sea bream [25]. However, these authors didn't give any explanation on this observed phenomenon. We inferred that low dietary P content stimulated the active transportation of P in the intestine and thus increased P absorption. 

Effects of dietary P level on growth performance were reported in many fish species. Improved growth was seen in common carp [26], juvenile haddock [2], juvenile silver perch [27], and juvenile black sea bream [26]. However, other studies reported that dietary P level did not change the growth of sea bass [28] and Atlantic salmon [29]. Animal growth depends on several factors, including age, stage of development, diet composition, duration of experiment, health, and rearing condition. Young animals were more sensitive to nutrient deficiency than those at a later stage of development [2]. In the current study, juvenile yellow catfish (average initial body weight was about 3 g) were sensitive to dietary P content and showed different growth performance under different dietary P treatments. However, the improved growth performance was only observed until the dietary available P level reached 0.81%, indicating that juvenile yellow catfish needed about 0.8% dietary available P to obtain maximum growth. There were several reports about catfish dietary available P requirements based on channel catfish (*Ictalurus punctatus*). Andrews et al. [18] reported that the P requirement of catfish is 0.8 percent of available P in practical-type diets. However, Lovell [19] and Wilson et al. [20] reevaluated the P requirement using chemically defined diets and estimated it to be approximately 0.42 percent available P. NRC [1] recommended the appropriate dietary available P to be 0.45 percent for channel catfish. However, in the present study, this small omnivorous fish gained highest growth at 0.81% dietary available P. The difference between yellow catfish and channel catfish was probably due to the species specific, different growth rate, and ingredients content of the diets or the culture system. 

Dietary P level had no effects on fish whole body moisture and crude protein content in the present study, which was similar to findings in red sea bream [30] and European sea bass [31]. However, crude lipid content decreased with increasing level of dietary P in our experiment. An inverse relationship between whole body crude lipid content and dietary P was also reported in channel catfish [32], red sea bream [30, 33], common carp [34], juvenile haddock [2], Japanese sea bass [35], large yellow croaker [36], and juvenile black sea bream [25]. Since P plays an important role in energy metabolism and lipid beta oxidation, P deficiency will cause cellular hypoxia and thus produce more lipid. The reduction in the lipid content of the fish fed higher levels of P may be due to enhancement of P oxidation of fatty acids, increase in glycogenesis or some type of repartitioning effect, resulting in increased deposition of protein and reduced deposition of lipid [32]. 

The whole-body or vertebrae ash and P levels had been commonly used as indicators of dietary P status in fish nutrition studies [4, 25, 36–38]. Signs of P deficiency are poor bone mineralization and bad growth performance. Dietary available P levels greatly affected the ash, calcium, and P contents both of the whole body and vertebrae of carp [39]. In our study, whole-body ash, vertebrae, ash and vertebrae P content of juvenile yellow catfish increased until dietary available P level reached 1.31% and then decreased at the 1.57% dietary available P level. Quadratic analysis based on vertebrae P contents indicated maximum vertebrae P at a dietary available P of 1.21%, higher than the requirement of 0.81% dietary available P for maximum growth. Similar findings were reported in large yellow croaker [36] and black sea bream [25]. The minimum available P requirement for maximum growth of large yellow croaker was 0.70%; however, based on the P content in either vertebrae or whole body, the requirements were 0.89% and 0.91%, respectively. In black sea bream, maximum weight gain was obtained at dietary available P concentration of 0.55%; however, quadratic analysis based on P content in vertebrae indicated that the requirement was 0.87%. The dietary P requirement for bone mineralization was usually higher than that for maximum growth [32, 40]. Differences among empirically established P requirements may be due in part to differences in the choice of response variable used [41]. The requirement must depend on what is considered a physiologically, economically, or environmentally important trait [32]. Thus, in the present study, if growth rate is used as a marker the requirement is 0.8%, but if maximum vertebrae P content is applied the requirement is 1.2%. If the dietary available P level is higher than 0.8% the growth is reduced. Considering the economic cost and environmental cost, dietary available P level of 0.8% is recommended for young yellow catfish.

Only a few preliminary researches have been reported about the roles of minerals in the antioxidant system of aquatic animals, including selenium [42], iron [43], and zinc [44], no research concerns the role of phosphorus in antioxidant defense systems in fish. Since P is important in energy metabolism and lipid beta oxidation, lipid peroxidation is supposed to be susceptible to dietary P level. In order to elaborate the basic oxidative functional responses of yellow catfish under different dietary P treatments so to determine whether they could be used as indicators of the stimulatory activity of dietary P, we measured hepatic SOD, CAT, and GPx activities and the content of MDA. We found hepatic SOD, CAT, and MDA contents except GPx activities were significantly affected by dietary available P level. Unlike in plant, no antioxidant responses were observed in low P groups (0.33% and 0.56%) compared to high P groups (1.57%). 

The effect of oral P exposure was reported in the form of white phosphorus [45]. LD_50_ values of 4.82 mg kg^−1^ and 4.85 mg kg^−1^ were reported for female and male mice, respectively [46]. Fish fed with the highest dietary available P (1.57%) had the highest hepatic SOD and CAT activities and highest MDA content. The SOD in fish is known to be induced in those tissues that are suffering from oxidative stress [47]. Ou et al. [48] claimed that the induction of these antioxidant enzymes is an important protective mechanism to minimize cell oxidative damage in unfavorable environments. In our study, high-level dietary P induced the antioxidative responses in juvenile yellow catfish. It is possible that the fish in this study were under an oxidative stress at the high levels of dietary available P which no longer served as a required nutrient but tended to be a stressor. Since SOD and CAT play the major role in the process of eliminating the free radicals [12], hepatic SOD and CAT were upregulated in juvenile yellow catfish in order to reduce free radicals produced by high dietary P. However, upregulated levels of SOD and CAT were not enough to eliminate the free radicals in fish fed 1.57% dietary available P, which led to the accumulation of the final product of lipid peroxidation, and finally the damage of cell membrane could not be restored in this group of fish.

## 5. Conclusions

This was the first study to show that dietary available P level affected hepatic antioxidant responses in fish. The current study indicated that the decrease of dietary available P increased the efficiency of P utilization and the body lipid content. However, vertebrae P also decreased, which could affect the bone mineralization of juvenile yellow catfish. Juvenile yellow catfish were subjected to oxidant damage and could not be reduced or eradicated by their own antioxidant defense system under the condition of high dietary P content. Therefore, as an essential nutrient for growth, bone mineralization, and maintaining the antioxidant property of this species, it is very important to optimize the dietary P content. In order to achieve the maximum growth rate, the optimal dietary available P of juvenile yellow catfish were estimated to be 0.81%. Based on maximum vertebrae P content, the optimal dietary available P of juvenile yellow catfish were estimated to be 1.21%.

## Figures and Tables

**Table 1:** Formulation and chemical composition of the experimental diets (g kg^−1^ dry matter).

Ingredients	Diets					
	1	2	3	4	5	6
Casein	350.0	350.0	350.0	350.0	350.0	350.0
Blood meal	100.0	100.0	100.0	100.0	100.0	100.0
Gelatin	20.0	20.0	20.0	20.0	20.0	20.0
Squid meal	70.0	70.0	70.0	70.0	70.0	70.0
Wheat flour	210.0	210.0	210.0	210.0	210.0	210.0
Fish oil	60.0	60.0	60.0	60.0	60.0	60.0
Soy bean oil	30.0	30.0	30.0	30.0	30.0	30.0
Vitamin premix^1^	5.0	5.0	5.0	5.0	5.0	5.0
Mineral premix (P free)^2^	27.5	27.5	27.5	27.5	27.5	27.5
Vitamin C	5.0	5.0	5.0	5.0	5.0	5.0
Choline chloride	2.5	2.5	2.5	2.5	2.5	2.5
Cr_2_O_3_	10.0	10.0	10.0	10.0	10.0	10.0
Ca(H_2_PO_4_)_2_·H_2_O	0.0	12.0	24.0	36.0	48.0	60.0
*α*-cellulose	110.0	98.0	86.0	74.0	62.0	50.0

Chemical composition (per kg dry matter)						
Moisture (g)	83.9	98.6	78.3	71.0	92.5	99.6
Crude protein (g)	455.9	452.9	447.5	446.1	457.5	444.1
Crude lipid (g)	62.8	62.6	65.4	60.1	63.3	62.7
Ash (g)	48.2	52.3	59.1	69.6	77.0	87.0
Total phosphorus (g)	3.8	6.3	8.9	12.2	14.2	17.1
Available phosphorus (g)	3.3	5.6	8.1	11.5	13.1	15.7

^1^Vitamin Premix was bought from Fulong Feed Company in Wuhan (Hubei province, China) and contained the following vitamins (mg kg^−1^ feed): vitamin K_3_, 20; niacin, 250; riboflavin, 50; pyridoxine, 25; thiamin, 25; D-calcium pantothenate, 100; biotin, 1.5; foliacin, 10; vitamin B_12_, 0.125; ascorbic acid, 250; inositol, 750; (IU kg^−1^ feed): vitamin A, 17500; vitamin D_3_, 2500; vitamin E, 175.

^2^Mineral premix (P free) contained the following minerals (mg kg^−1^ feed): NaCl, 275; MgSO_4_·7H_2_O, 4125; Na_2_SO_4_, 6270; K_2_SO_4_, 5638; CaCl_2_·2H_2_O, 3218; FeSO_4_, 688; Calcium lactate, 963; ZnSO_4_·7H_2_O, 97; MnSO_4_·4H_2_O, 45; CuSO_4_·5H_2_O, 8.53; CoSO_4_, 0.28; KI, 0.83.

**Table 2:** Effects of dietary available P level on apparent digestibility coefficient (ADC), P utilization efficiency, and feces P content in yellow catfish fed experimental diets for eight weeks (Mean ± SE, *n* = 3).

Available P in diet (%)	0.33	0.56	0.81	1.15	1.31	1.57
Dry matter (ADCd, %)	87.21 ± 2.94	83.21 ± 2.58	87.17 ± 1.37	89.29 ± 1.29	88.08 ± 2.69	88.21 ± 0.04
Protein (ADCp, %)	98.22 ± 0.41	96.89 ± 0.43	97.48 ± 0.13	97.80 ± 0.36	97.38 ± 0.65	97.47 ± 0.06
P (ADCph, %)	87.01 ± 3.40^b^	89.43 ± 1.26^bc^	90.47 ± 0.67^abc^	94.18 ± 0.50^a^	92.57 ± 1.59^ac^	92.06 ± 0.20^ac^
Efficiency of P utilization (%)	77.46 ± 7.45^a^	57.99 ± 5.37^ab^	54.45 ± 3.81^b^	46.38 ± 7.43^b^	37.51 ± 3.41^b^	45.18 ± 10.30^b^
Feces P (g kg^−1^ dry matter)	0.38 ± 0.01^d^	0.40 ± 0.02^d^	0.67 ± 0.03^c^	0.67 ± 0.03^c^	0.89 ± 0.01^b^	1.15 ± 0.03^a^

Note: values with different letters within the same row are significantly different (*P* < 0.05).

**Table 3:** The growth performance of yellow catfish under different dietary treatments for eight weeks (Mean ± SE, *n* = 3).

Available P in diet (%)	0.33	0.56	0.81	1.15	1.31	1.57
IBW (g)	3.02 ± 0.05	3.10 ± 0.03	3.03 ± 0.09	3.00 ± 0.07	3.14 ± 0.06	3.23 ± 0.01
FBW (g)	6.08 ± 0.09^bc^	6.44 ± 0.08^b^	7.51 ± 0.36^a^	5.45 ± 0.23^c^	6.19 ± 0.36^bc^	6.58 ± 0.21^b^
SGR (% day^−1^)	1.25 ± 0.01^bc^	1.31 ± 0.02^b^	1.62 ± 1.10^a^	1.06 ± 0.04^c^	1.21 ± 0.08^bc^	1.27 ± 0.06^b^
FR (% BW day^−1^)	1.74 ± 0.08^b^	1.70 ± 0.02^b^	1.93 ± 0.02^a^	1.46 ± 0.06^c^	1.57 ± 0.04^c^	1.54 ± 0.01^c^
FE (%)	64.40 ± 1.81	68.57 ± 1.07	73.04 ± 3.69	64.92 ± 4.05	69.45 ± 2.59	76.31 ± 3.83
PER (%)	1.36 ± 0.04^b^	1.45 ± 0.04^b^	1.56 ± 0.11^ab^	1.35 ± 0.10^b^	1.45 ± 0.04^b^	1.70 ± 0.09^a^
PRE (%)	17.63 ± 0.94	19.26 ± 3.29	21.33 ± 1.72	21.75 ± 1.02	19.91 ± 1.96	21.59 ± 2.61
Survival rate (%)	92.22 ± 0.56	91.67 ± 1.67	89.44 ± 2.00	90.56 ± 2.00	92.78 ± 2.42	96.11 ± 0.56

Note: IBW: initial body weight; FBW: final body weight; SGR: specific growth rate; FR: feeding rate; FE: feed efficiency; PER: protein efficiency ratio; PRE: protein retention efficiency.

Values with different letters within the same row are significantly different (*P* < 0.05).

**Table 4:** Effects of dietary available P level on body composition of yellow catfish for eight weeks (Mean ± SE, *n* = 3).

Available P in diet (%)	Initial	0.33	0.56	0.81	1.15	1.31	1.57
Whole body (% of wet weight)							
Moisture	76.95 ± 0.60	74.20 ± 1.62	75.73 ± 1.48	74.26 ± 0.78	74.44 ± 0.99	75.07 ± 1.00	76.89 ± 1.35
Crude protein	12.79 ± 0.40	12.93 ± 0.50	13.01 ± 1.00	13.28 ± 0.23	14.10 ± 0.65	13.24 ± 0.68	12.75 ± 0.67
Crude lipid	3.83 ± 0.21^c^	6.49 ± 0.70^a^	4.57 ± 0.60^bc^	5.47 ± 0.61^ab^	4.43 ± 0.26^bc^	4.44 ± 0.25^bc^	3.62 ± 0.43^c^
Ash	3.36 ± 0.13^bd^	3.27 ± 0.16^d^	3.63 ± 0.12^bd^	3.97 ± 0.23^abc^	4.31 ± 0.19^a^	4.46 ± 0.34^a^	4.14 ± 0.17^ab^
Body P	0.64 ± 0.07	0.63 ± 0.02	0.66 ± 0.02	0.71 ± 0.04	0.81 ± 0.05	0.80 ± 0.04	0.81 ± 0.09
Body Ca	0.26 ± 0.02^c^	0.30 ± 0.03^bc^	0.33 ± 0.01^ab^	0.37 ± 0.03^a^	0.38 ± 0.01^a^	0.38 ± 0.01^a^	0.38 ± 0.01^a^

Vertebrae (P and Ca, g kg^−1^; ash, % of dry matter)							
P	72.00	59.58 ± 2.92^c^	64.66 ± 0.30^bc^	66.99 ± 1.74^ab^	69.28 ± 2.07^ab^	72.26 ± 1.72^a^	67.46 ± 0.86^ab^
Ca	38.78	28.51 ± 1.19^b^	34.09 ± 0.93^a^	33.67 ± 2.13^a^	34.15 ± 0.79^a^	35.67 ± 2.36^a^	35.84 ± 0.96^a^
Ash	38.35	28.49 ± 1.10^c^	34.63 ± 0.21^b^	35.60 ± 0.98^b^	37.11 ± 0.86^ab^	39.08 ± 0.68^a^	36.58 ± 0.48^ab^

Note: values with different letters within the same row are significantly different (*P* < 0.05).

**Table 5:** Effects of dietary available P level on hepatic biochemical indicators of yellow catfish for eight weeks (Mean ± SE, *n* = 3).

% P in diet	0.33	0.56	0.81	1.15	1.31	1.57
SOD (U mgpro^−1^)	33.94 ± 2.27^c^	37.09 ± 4.07^bc^	46.19 ± 5.37^ab^	37.74 ± 0.69^bc^	39.98 ± 3.47^bc^	56.46 ± 4.92^a^
CAT ( U mgpro^−1^)	4.87 ± 0.54^b^	4.75 ± 0.44^b^	5.68 ± 0.42^ab^	4.59 ± 0.27^b^	4.79 ± 0.32^b^	6.97 ± 0.63^a^
MDA (nmol mgpro^−1^)	0.48 ± 0.06^b^	0.50 ± 0.04^b^	0.77 ± 0.06^a^	0.45 ± 0.03^b^	0.54 ± 0.07^b^	0.89 ± 0.11^a^
GSH-Px (U)	2.02 ± 0.44	2.66 ± 0.61	1.89 ± 0.50	1.47 ± 0.58	2.18 ± 0.33	2.43 ± 0.56

Note: values in the same row with different letters are significantly different (*P* < 0.05).

SOD: superoxide dismutase; CAT: catalase; GSH-Px: glutathione peroxidase; MDA: malonaldehyde.
